# The little shrimp that could: phylogeography of the circumtropical *Stenopus hispidus* (Crustacea: Decapoda), reveals divergent Atlantic and Pacific lineages

**DOI:** 10.7717/peerj.4409

**Published:** 2018-03-06

**Authors:** ‘Ale‘alani Dudoit, Matthew Iacchei, Richard R. Coleman, Michelle R. Gaither, William E. Browne, Brian W. Bowen, Robert J. Toonen

**Affiliations:** 1 Hawai‘i Institute of Marine Biology, School of Ocean and Earth Science and Technology, University of Hawai‘i at Mānoa, Kāne‘ohe, HI, United States of America; 2 Department of Zoology, University of Hawai‘i at Mānoa, Honolulu, HI, United States of America; 3 Department of Oceanography, School of Ocean and Earth Science and Technology, University of Hawai‘i at Mānoa, Honolulu, HI, United States of America; 4 Department of Natural Science, Hawai‘i Pacific University, Kāne‘ohe, HI, United States of America; 5 Department of Biology, University of Miami, Coral Gables, FL, United States of America; 6 Current affiliation: Department of Biology, University of Central Florida, Orlando, FL, United States of America

**Keywords:** Coral reefs, Cytochrome *c* oxidase subunit I, Population structure, Cosmopolitan distribution, Marine biogeography

## Abstract

The banded coral shrimp, *Stenopus hispidus* (Crustacea: Decapoda: Stenopodidea) is a popular marine ornamental species with a circumtropical distribution. The planktonic larval stage lasts ∼120–253 days, indicating considerable dispersal potential, but few studies have investigated genetic connectivity on a global scale in marine invertebrates. To resolve patterns of divergence and phylogeography of *S. hispidus*, we surveyed 525 bp of mitochondrial cytochrome *c* oxidase subunit I (COI) from 198 individuals sampled at 10 locations across ∼27,000 km of the species range. Phylogenetic analyses reveal that *S. hispidus* has a Western Atlantic lineage and a widely distributed Indo-Pacific lineage, separated by sequence divergence of 2.1%. Genetic diversity is much higher in the Western Atlantic (*h* = 0.929; *π* = 0.004) relative to the Indo-Pacific (*h* = 0.105; *π* < 0.001), and coalescent analyses indicate that the Indo-Pacific population expanded more recently (95% HPD (highest posterior density) = 60,000–400,000 yr) than the Western Atlantic population (95% HPD = 300,000–760,000 yr). Divergence of the Western Atlantic and Pacific lineages is estimated at 710,000–1.8 million years ago, which does not readily align with commonly implicated colonization events between the ocean basins. The estimated age of populations contradicts the prevailing dispersal route for tropical marine biodiversity (Indo-Pacific to Atlantic) with the oldest and most diverse population in the Atlantic, and a recent population expansion with a single common haplotype shared throughout the vast Indian and Pacific oceans. In contrast to the circumtropical fishes, this diminutive reef shrimp challenges our understanding of conventional dispersal capabilities of marine species.

## Introduction

Despite the seemingly broad continuity of the world’s oceans, relatively few marine species achieve and maintain circumtropical distributions. To do so requires high dispersal potential to sustain gene flow across large geographic expanses, and a tolerance for a broad range of habitats and environmental conditions. In a recent survey of fishes, Gaither et al. (2015a) found that fewer than 1% have circumtropical distributions. Seventy percent of these cosmopolitan fishes are species that thrive in the relatively continuous, and comparatively environmentally stable habitat of the bathypelagic zone (>200 m depth). Many other circumtropical fishes are large pelagic species, such as billfishes, sharks, and tunas, with adult ranges spanning whole ocean basins and broad depth distributions (Castro et al., 2007; Collette et al., 2011). No parallel synthesis has been conducted for invertebrates, and the few examples of circumtropical invertebrates we found in the literature were either pelagic or mesopelagic species (e.g., Norris, 2000; Van der Spoel & Heyman, 1983), associated with open ocean currents (e.g., Trickey, Thiel & Waters, 2016; Willan, 1979), or fouling organisms of cryptogenic origin (e.g., Foster & Willan, 1979; Jones, 1992).

For benthic-associated taxa, there are additional challenges to maintaining a global distribution; only 7% (18 species) of the circumtropical fishes occupy shallow benthic habitats (Gaither et al., 2015a). Although impenetrable barriers to dispersal are relatively rare in the sea, there are eight major biogeographic barriers that limit most species’ distributions in some portion of their range (Briggs, 1974; Briggs & Bowen, 2012; Toonen et al., 2016). All of the tropical barriers interrupt benthic habitat continuity, whether through sharp oceanographic or ecological gradients (Amazon River, Benguela Current, and Red Sea Barriers), vast expanses of open ocean lacking shallow water habitat (East Pacific and Mid-Atlantic Barriers), or temporal changes in terrestrial habitat emergence due to fluctuations in ocean depth (Isthmus of Panama, Red Sea, and Sunda Shelf Barriers; see Fig. 1 in Toonen et al., 2016). These barriers often form the range edges for sister species that diverged in allopatry (e.g., Barber et al., 2000; Bernardi, Findley & Rocha-Olivares, 2003; Bowen et al., 2016; Briggs, 1973; Cowman & Bellwood, 2013a; DiBattista et al., 2013; Floeter et al., 2008; Gaither et al., 2010; Hodge & Bellwood, 2016; Marko, 2002; Mayr, 1954; Rocha, 2003). In addition to these historical biogeographic barriers, present day environmental gradients, habitat availability, and intrinsic biological traits have all contributed to the distribution of marine fauna. These biogeographic provinces, defined as areas where >10% of the species are endemic (Briggs, 1974), have been formulated, revised, and studied for more than a century (reviewed by Briggs & Bowen, 2012; Toonen et al., 2016). Each provincial region hosts recent evolutionary innovation and/or relictual ranges of ancient lineages (Cowman & Bellwood, 2013b). These provincial frameworks have been refined over time, due to the continuing accrual of species distribution data, and more recently, molecular techniques that have redefined some species complexes (e.g., Bowen, Karl & Pfeiler, 2007; Durand et al., 2012; Forsman et al., 2015), including pelagic and bathypelagic taxa (Andrews et al., 2014; Goetze, 2003; Hirai, Tsuda & Goetze, 2015; Miya & Nishida, 1997). Additional biogeographic frameworks based on species presence/absence data (Kulbicki et al., 2013), coral biogeography (Veron et al., 2015), and nested marine ecoregions (Spalding et al., 2007) have enabled the testing of hypotheses regarding the origin and diversification of marine species (e.g., Cowman et al., 2017).

The accumulation of molecular data from a diversity of taxa has advanced the longstanding goal of understanding whether and where concordance exists between population separations within species, interspecific species divergences, and biogeographic barriers in the sea (Bowen et al., 2016; Karl & Avise, 1992; Lessios et al., 1999; Palumbi, 1996). Comparative molecular approaches using multiple taxa have demonstrated concordance, for example, across the Red Sea Barrier (DiBattista et al., 2013), the Indo-Pacific Barrier (Gaither & Rocha, 2013), the East Pacific Barrier (Lessios & Robertson, 2006), and the Benguela Barrier (Teske et al., 2011), in addition to elucidating shared population boundaries within single biogeographic provinces (e.g., Barber et al., 2011; DiBattista et al., 2017; Pope et al., 2015; Toonen et al., 2011). The comparative approach has also enabled the deciphering of probable mechanisms of differentiation across individual biogeographic barriers and within provincial regions (e.g., Hickerson, Meyer & Moritz, 2006; Keith et al., 2015; Luiz et al., 2012; Selkoe et al., 2014). However, while many marine species range edges are clustered around biogeographic barriers, and some span at least one or more barriers, relatively few species cross multiple boundaries without divergence, and molecular surveys of these broadly distributed benthic coral reef taxa have been relatively rare (Keyse et al., 2014). Coral reef species that span more than two biogeographic barriers often reveal population genetic separations at these barriers (Bowen et al., 2016; Iacchei et al., 2016). Although still few in number, these results generally support concordance between intraspecific and interspecific divergences, and affirm that population separations can be a starting point for speciation (Bowen et al., 2013; Bowen et al., 2016). Targeting additional species with maximal range sizes, and in particular those that span multiple ocean basins, may illuminate the factors that define species distributions and the strength and pervasiveness of biogeographic barriers. One approach is to investigate species that somehow extend across multiple biogeographic provinces compared to closely related species that do not.

**Figure 1:**
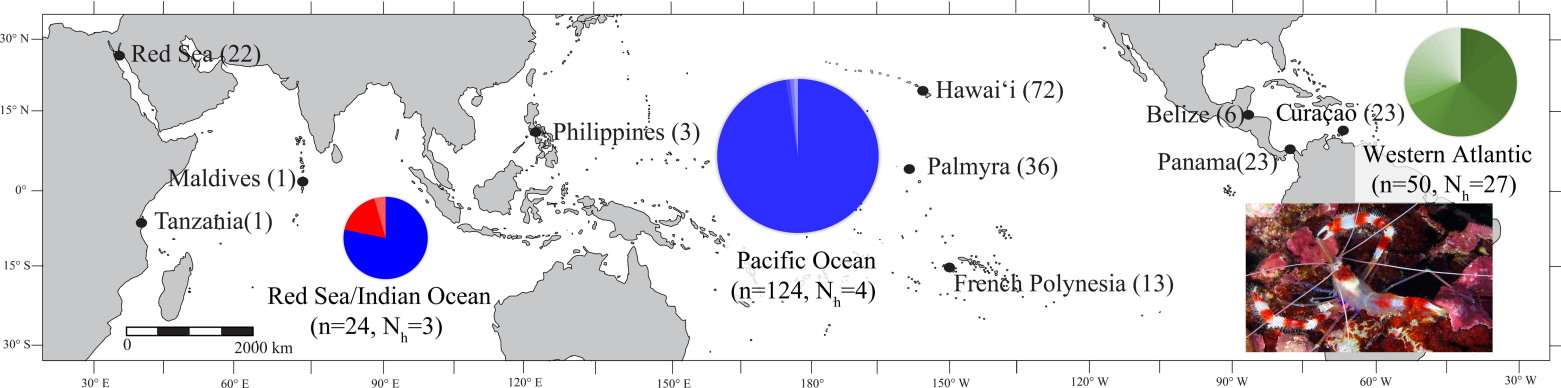
Map of the sample locations for *Stenopus hispidus* depicting the number of individuals (*n*) and number of haplotypes (*N*_*h*_) sampled at each location. Pie charts are shown for each region (Red Sea, Indian Ocean, Pacific Ocean, and Western Atlantic) representing the proportion of haplotypes resolved for a 525 bp mitochondrial COI sequence fragment for individuals sampled in that region. The size of the pie chart is proportional to number of individuals sampled in that region. Each color in the pie charts represents a unique haplotype resolved in that region, and the size of the color wedges represent the proportion of sampled individuals in the region that contained that haplotype. Haplotypes in the Red Sea/Indian Ocean are represented in red and blue, with the blue haplotypes also found in the Pacific Ocean. Haplotypes of the Western Atlantic are represented in green and are not shared with any other region. Photo credit: Keoki Stender.

Here we investigate the phylogeography of the banded coral shrimp *Stenopus hispidus* (Olivier, 1811) to examine the influence of biogeographic boundaries on this circumtropical species. Within the genus *Stenopus* (family Stenopodidae), there are 11 species, 10 of which are range-limited by biogeographic barriers. However, the family Stenopodidae is an understudied group without a detailed phylogenetic analysis of the current taxonomy. A recent phylogeny indicates that the family is non-monophyletic, and that *Stenopus* is a polyphyletic genus, with at least two species, *S. earlei* and *S. goyi*, likely to be reclassified into *Juxtastenopus* (Chen et al., 2016). Regardless of generic rearrangements, among currently recognized species of Stenopodidae, only the banded coral shrimp *S. hispidus* inhabits tropical coral reefs throughout the Pacific, Indian, and Atlantic Oceans (Fig. 1). This distribution crosses three of the most substantial biogeographic barriers on the planet: the Sunda Shelf Barrier (Briggs, 1974), the break between the Red Sea and the Indian Ocean (DiBattista et al., 2013; DiBattista et al., 2016; Klausewitz, 1989), and the cold-upwelling Benguela Current along the South-West coast of Africa (Garzoli & Gordon, 1996; Hutchings et al., 2009). A broad depth range (1–210 m; Limbaugh, Pederson & Chace, 1961), generalist habitat, and long pelagic larval duration (PLD) (∼253 days; De Castro & Jory, 1983; Goy, 1990) have all been hypothesized as potential life history characteristics enabling the extensive distribution of *S. hispidus*. To date, however, no studies have examined the phylogeography of this species to test whether *S. hispidus* maintains a single global population, or if major biogeographic barriers limit gene flow. Here, we present the first phylogeographic study of this species, to investigate whether biogeographic barriers that limit the distribution of congeneric species also act as barriers to gene flow in *S. hispidus*.

## Materials and Methods

### Sample collection

*Stenopus hispidus* (*N* = 198) were collected from ten locations across the >27,000 km species range (Fig. 1) (Papahānaumokuākea Marine National Monument (PMNM) collection permit no. PMNM-2008-047, PMNM-2009-032, PMNM-2010-037, PMNM-2011-041, PMNM-2012-049). Collections were made via snorkeling, and on both open circuit (OC) and closed circuit (CCR) SCUBA (Ambient Pressure Diving Ltd., Helston, UK). Where possible, specimens were collected nonlethally as single legs or claws. Tissue samples were preserved in saturated salt DMSO buffer (Seutin, White & Boag, 1991) or 95% ethanol and stored at room temperature.

### Mitochondrial DNA sequencing

Genomic DNA was isolated using either the OMEGA Bio-tek extraction kit (OMEGA Bio-tek, Inc., Norcross, GA, USA) following manufacturer’s instructions or the ‘HotSHOT’ protocol (Meeker et al., 2007) and was stored at −20 °C. A fragment of the mitochondrial cytochrome *c* oxidase subunit I (COI) gene was amplified by the polymerase chain reaction (PCR) using the primers LCO1490 and HCO2198 (Folmer et al., 1994). PCR amplifications were conducted in a final reaction volume of 15 µl, consisting of 2–15 ng of template DNA, 0.073 µM of each primer, 7.5 µl of 2X BioMix Red (Bioline Inc., Springfield, NJ, USA), and deionized water to volume. PCR conditions consisted of an initial denaturation at 95 °C for 1 min; 35 cycles of denaturation at 95 °C for 1 min, annealing at 40 °C for 40 s, extension at 72 °C for 90 s; and a final extension at 72 °C for 7 min.

PCR products were visualized using a 1.5% agarose gel with GelStar™ (Cambrex Bio Science Rockland, MA, USA). Amplification products were prepared for sequencing using 0.75 units of Exonuclease I and 0.5 units of Fast Alkaline Phosphatase (ExoFAP, Thermo Fisher Scientific, Waltham, MA, USA) per 5 µl PCR product, and incubated at 37 °C for 30 min, followed by deactivation at 85 °C for 15 min. Purified DNA fragments were sequenced in the forward direction with fluorescently-labeled dideoxy terminators on an ABI 3730XL Genetic Analyzer (Applied Biosystems, Foster City, CA, USA) at the University of Hawai‘i Advanced Studies of Genomics, Proteomics and Bioinformatics sequencing facility. Unique or ambiguous sequences were sequenced in the reverse direction to assure correct base calls. Sequences were aligned, edited, and trimmed to a uniform length using GENEIOUS PRO 6.1.8 (Biomatters Ltd., Auckland, New Zealand). There were no indels, frameshift mutations, or stop codons in the alignment. Sequences were deposited in Genbank (MG819599–MG819631) while associated metadata can be found in the open access repository GeOMe (Deck et al., 2017).

### Phylogenetic analyses

Sequences were aligned with GENEIOUS PRO. We used jModelTest 0.1.1 (Posada, 2008) to determine that HKY + I model of evolution is the best fit for our dataset using the Bayesian Information Criterion (BIC). For analyses that were not able to incorporate the HKY + I model of sequence evolution, we applied the model highest-ranked using the BIC that could be implemented in the program. All phylogenetic reconstructions were rooted with *Stenopus pyrsonotus* (Genbank, MG819632), *Stenopus zanzibaricus* (Bold, MBMIA506-06), and *Stenopus scutellatus* (Genbank, MG819633). For some reconstructions, we also used additional outgroups that are more distantly related, *Synalpheus* sp. (Bold, CRM118-10), and *Alpheus bellulus* (Bold, AMINV055-08) (Fig. S1).

A maximum-likelihood phylogeny was produced using the HKY model of evolution and a neighbor-joining phylogeny was constructed using the Tamura & Nei model of evolution in the program MEGA 6.06 (Tamura et al., 2013). Branch support values for both maximum-likelihood and neighbor-joining phylogenies were calculated using 1,000 bootstrap replicates. We also calculated mean genetic distance (*d*) between lineages in DNASP 5 (Librado & Rozas, 2009).

A Bayesian inference phylogenetic network was computed in BEAST 2.2.0 (Drummond et al., 2012) using the HKY + I model of evolution. To estimate the time to most recent common ancestor (TMRCA), we formatted the data with BEAUTI 2.2.0, and used a Bayesian Markov Chain Monte Carlo (MCMC) approach in BEAST (Drummond et al., 2012).

Since divergence rates do not exist for the genus *Stenopus*, due to a lack in the fossil record, we used established divergence rates (8.7%) from a sister genus *Alpheus* (Lessios, 2008; Shi et al., 2012) to calculate a molecular rate of evolution for *S. hispidus*. Assuming the closure of the Isthmus of Panama was ∼3 Mya (Lessios, 2008; Marko, 2002; Marko & Moran, 2009; O’Dea et al., 2016), we used the estimated divergence rate of 1.45%/My for *S. hispidus* here. Our TMRCA was constructed with a strict clock of 1.45% per million years between lineages and using a coalescent tree prior to assuming exponential growth. We used default priors under the HKY model and ran 10 million generations, with trees saved every 10,000 generations, and the first 10% of trees discarded as burn-in. Ten independent runs were computed to ensure convergence and log files were combined using the program TRACER 1.6.0 (http://tree.bio.ed.ac.uk/software/tracer/). A maximum clade credibility tree was constructed using TREE ANNOTATOR 1.6.0. The program FIGTREE 1.4.2 (http://tree.bio.ed.ac.uk/software/figtree/) was used to visualize the phylogeny.

### Phylogeographic analyses

Haplotype (*h*) and nucleotide diversity (*π*) were calculated using the program ARLEQUIN 3.5 (Excoffier & Lischer, 2010). Region-specific haplotype diversities were also calculated for the Red Sea and Indian Ocean combined (due to small sample size in the Indian Ocean), the Pacific Ocean, and the Western Atlantic to facilitate regional comparisons. A median-joining network was constructed to visualize the relationships among haplotypes, using the program NETWORK 4.6 (Bandelt, Forster & Rohl, 1999) with default settings.

An analysis of molecular variance (AMOVA) was used to test for hierarchical population genetic structure among regions in ARLEQUIN using 50,000 permutations to test for significance. Locations where sample sizes were <5 (Philippines, Maldives, Tanzania) were excluded from population-level analyses but included in overall diversity estimates. Pairwise genetic differentiation (Φ_ST_) was calculated between each pair of sampling locations in ARLEQUIN using the *TrN* (Tamura & Nei, 1993) model of sequence evolution with a *Ti*∕*Tv* ratio of 3.47. Among the models that ARLEQUIN supports, this was the highest ranked model of sequence evolution using the BIC. Statistical significance of pairwise Φ_ST_ estimates were determined using 50,000 permutations and the false discovery rate was controlled according to Narum (2006) (adjusted *P* < 0.014). Finally, we investigated the correlation between pairwise genetic (Φ_ST_) and geographic (km) distance (log–log transformed), within ocean basins (Pacific Ocean, Red Sea/Indian Ocean, and Atlantic Ocean) using Mantel tests to test for significance (20,000 iterations) on the Isolation-by-Distance Web Service 3.16 (Jensen, Bohonak & Kelley, 2005).

## Results

### Phylogenetic analysis

**Figure 2:**
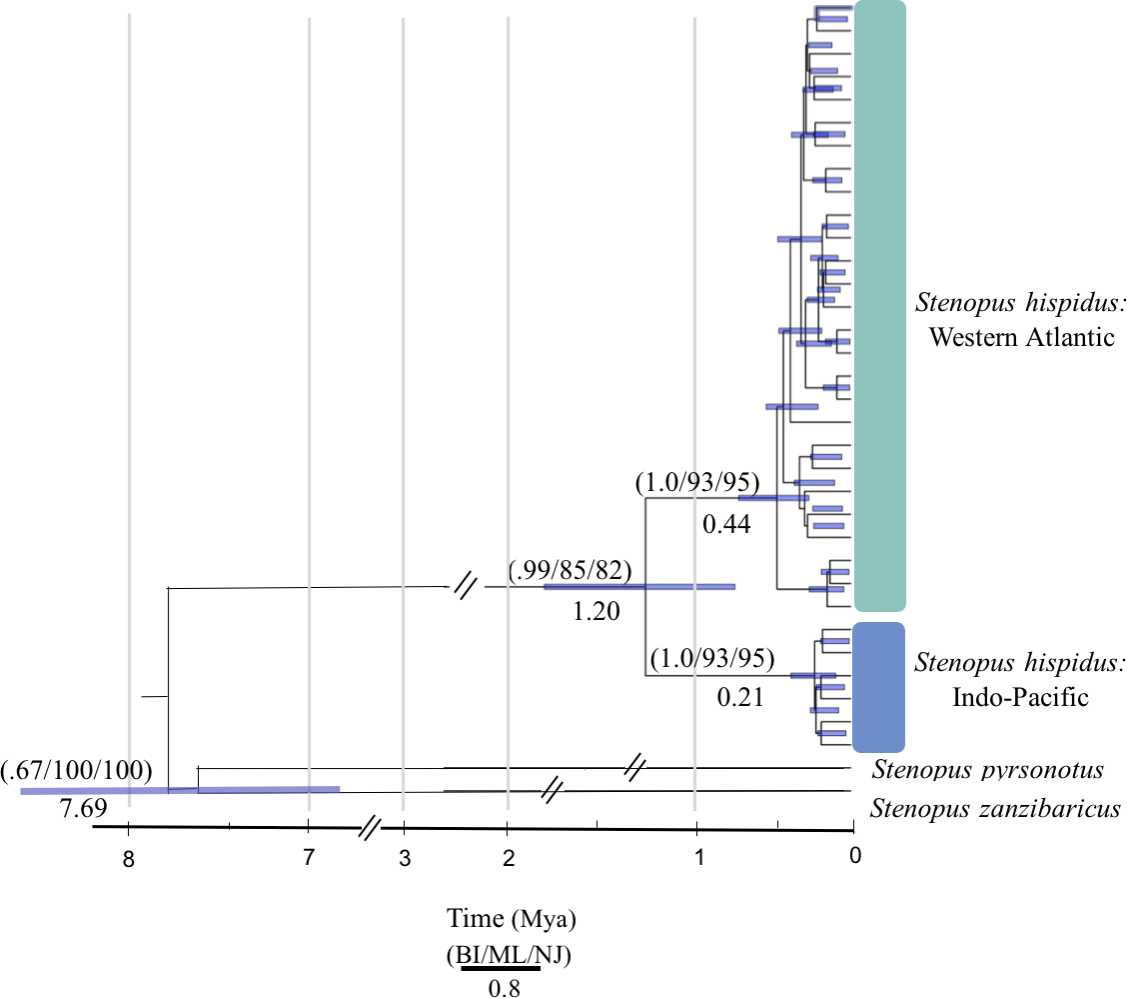
Molecular phylogenetic reconstruction of *Stenopus hispidus*. A rooted Bayesian tree based on 525 bp of cytochrome *c* oxidase subunit I (COI) with posterior probabilities from Bayesian Inference (BI), maximum likelihood bootstrap support (ML), and neighbor-joining bootstrap support (NJ). Two sister species (*S. pyrsonotus*, *S. zanzibaricus*) are represented as outgroups. The Western Atlantic lineage of *S. hispidus* is shown in green (*N* = 50; Curaçao, Belize, and Panama). The Indo-Pacific lineage of *S. hispidus* is shown in blue (*N* = 148; Hawai‘i, Palmyra Atoll, French Polynesia, Philippines, Maldives, Tanzania, and the Red Sea). Values below nodes are median node ages with 95% high posterior density intervals represented by blue node bars.

**Figure 3:**
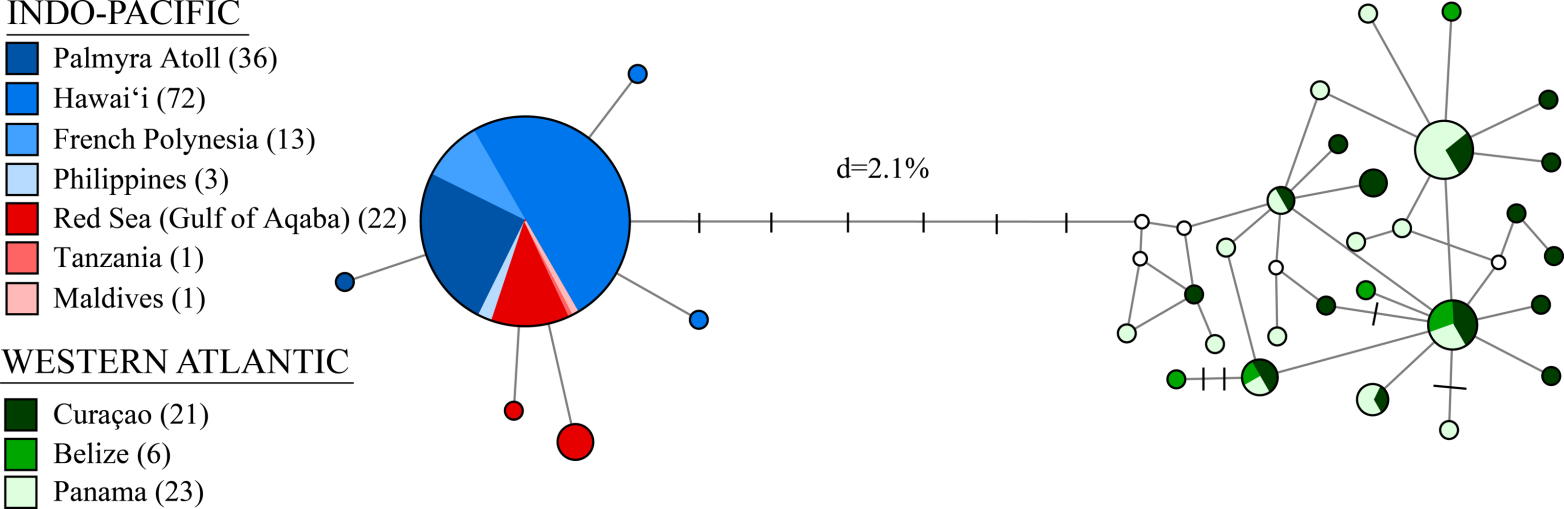
A median-joining network for 198 individuals of *Stenopus hispidus* based on 525 bp of the cytochrome *c* oxidase subunit I (COI) gene. Total numbers of individuals collected at each location are in parenthesis. Each circle represents a unique haplotype and its size is proportional to its total frequency. Lines and each black crossbar represent a single nucleotide change whereas open white circles indicate unsampled haplotypes. The network depicts two distinct lineages separated by seven mutational steps and a sequence divergence (*d*) of 2.1%.

We resolved a 525 bp fragment of COI for 198 individuals at 10 locations across the circumtropical species range. Phylogenetic analyses revealed two lineages that corresponded to a Western Atlantic lineage observed at Curaçao, Belize, and Panama and an Indo-Pacific lineage consisting of all sample sites in the Pacific Ocean, Red Sea and Indian Ocean (Bayesian posterior probability = 1.0; ML bootstrap = 93; NJ = 95) (Fig. 2). The average proportion of nucleotide substitutions per site between lineages was *d* = 2.1% (Fig. 3). Coalescent analysis indicated the TMRCA for the *S. hispidus* lineage to be 1.2 Mya (95% HPD = 710,000–1.8 Mya). The TMRCA for the Western Atlantic lineage was ∼500,000 years (95% HPD = 300,000–760,000 yrs), while the Indo-Pacific lineage was ∼200,000 years (95% HPD = 60,000–400,000 yrs).

### Phylogeographic analyses

Molecular indices are summarized in Table 1. We observed a total of 33 distinct haplotypes, with only six haplotypes found in the Indo-Pacific, despite more extensive sampling, and 27 haplotypes in the Western Atlantic (Table 1, Fig. 3). The Western Atlantic and Indo-Pacific shared no haplotypes, with the two closest haplotypes between these ocean basins separated by nine mutations, compared to five mutations across the entire Indo-Pacific (Fig. 3). Mean haplotype diversity (*h)* was 0.547 with a range from 0.055 to 0.962 (Table 1). Mean nucleotide diversity (*π*) was 0.005 with a range from <0.001 to 0.006 (Table 1). Genetic diversity was highest in the Western Atlantic (*h* = 0.929 and *π* = 0.005, with much lower diversity detected in the Indo-Pacific (*h* = 0.165 and *π* < 0.001) (Table 1). If we subdivide the Indo-Pacific by region, the Pacific Ocean had the lowest diversity (*h* = 0.048; *π* < 0.001; *N* = 124) and the Red Sea and Indian Ocean had the highest diversity (*h* = 0.385; *π* < 0.001; *N* = 24) (Table 1).

**Table 1:** Summary statistics of molecular diversity and coalescence times based on cytochrome *c* oxidase subunit I gene (COI) from 10 locations in the Western Atlantic and Indo-Pacific Oceans. Total number of individuals (*n*), number of haplotypes (*N*_*h*_), polymorphic sites (*ps*), haplotype diversity (*h*), nucleotide diversity (*π*), and effective haplotypes (*H*_*eff*_). Dashes represent incalculable values because *N*_*h*_ = 1. French Polynesia includes Mo’ore’a and Marquesas.

Sample location	*n*	*N*_*h*_	*ps*	*h*	*π*	*H*_*eff*_
**Pacific ocean**						
Hawai‘i	72	3	2	0.055	<0.001	1.058
Palmyra Atoll	36	2	1	0.056	<0.001	1.059
French Polynesia	13	1	–	–	–	
Philippines	3	1	–	–	–	
**Indian ocean**						
Maldives	1	1	–	–	–	
Tanzania	1	1	–	–	–	
**Red sea**						
Gulf of Aqaba	22	3	2	0.385	<0.001	1.626
**Western Atlantic**						
Curaçao	21	15	17	0.962	0.005	26.316
Belize	6	5	7	0.933	0.006	14.925
Panama	23	14	14	0.881	0.005	8.403
**Western Atlantic**	50	27	38	0.929	0.005	13.889
**Indo-Pacific**	148	6	5	0.165	<0.001	1.198
All samples	198	33	43	0.547	0.005	2.205

Global population structure was Φ_ST_ = 0.868 (*P* < 0.001). A regional analysis of molecular variance (AMOVA) indicates significant genetic differentiation among the Red Sea/Indian, Pacific, and Western Atlantic Ocean Basins (Φ_CT_ = 0.905; *P* = 0.008; Table 2). There were significant pairwise Φ_ST_ values between sampling locations in the Western Atlantic and the Indo-Pacific Oceans (Φ_ST_ = 0.859–0.976; *P* < 0.001 for all comparisons; Table 3). After correcting for false discovery rate, all pairwise comparisons remained significant (Table 3). Isolation by distance (IBD) was significant among ocean basins for the log–log transformed (*R*^2^ = 0.089, *P* = 0.03) data; however, we did not have enough sampling locations to test within ocean basins.

**Table 2:** Analysis of molecular variance (AMOVA) based on the cytochrome *c* oxidase subunit I (COI) for *Stenopus hispidus*. Φ-statistics among groups (Φ_CT_), *P*-values (*P*), percent of variation, degrees of freedom (d.f.), sum of squares (SS), and variance components (Var) for each biogeographical framework tested for *S. hispidus*. All regional comparisons are significant at *P* < 0.05. (RS/IO, Red Sea/Indian Ocean; PO, Pacific Ocean; WAO, Western Atlantic Ocean).

Among groups						
Among regions	Φ_CT_	*P*	% of variation	*d.f.*	SS	Var
RS/IO vs. PO	0.235	0.029	23.46	1	0.599	0.015
RS/IO and PO vs. WAO	0.930	0.008	93.05	1	368.088	4.916
RS/IO vs. PO vs. WAO	0.905	0.008	90.51	2	368.687	3.505

**Table 3:** Pairwise statistics for *Stenopus hispidus* for seven locations using a 525 base pair fragment of the cytochrome c oxidase subunit I gene (COI). Φ_ST_ is below the diagonal and the *P*-values are above the diagonal. Significant Φ_ST_ values are in bold. Locations that have fewer than three individuals sampled (Philippines, Maldives, and Tanzania) have been removed from this analysis. French Polynesia includes Mo’ore’a and the Marquesas.

	Sample location	Indo-Pacific				Western Atlantic		
		Hawai‘i	Palmyra Atoll	French Polynesia	Red Sea	Curaçao	Belize	Panama
Indo-Pacific	Hawai‘i	–	0.561	0.999	0.001	<0.001	<0.001	<0.001
	Palmyra Atoll	0.000	–	0.999	0.009	<0.001	<0.001	<0.001
	French Polynesia	−0.039	−0.035	–	0.272	<0.001	<0.001	<0.001
	Red Sea	**0.189**	**0.135**	0.070	–	<0.001	<0.001	<0.001
Western Atlantic	Curaçao	**0.942**	**0.910**	**0.859**	**0.871**	–	0.206	0.300
	Belize	**0.976**	**0.959**	**0.917**	**0.917**	0.030	–	0.059
	Panama	**0.942**	**0.911**	**0.863**	**0.874**	0.006	0.083	–

## Discussion

Our phylogeographic survey of the circumtropical banded coral shrimp, *Stenopus hispidus* reveals two divergent and reciprocally monophyletic mitochondrial lineages: a diverse lineage in the Western Atlantic and a depauperate Indo-Pacific lineage, predominated by a single common haplotype observed at every sample site throughout the Indo-Pacific and Red Sea. As expected for reciprocally monophyletic lineages, genetic structure was high between the Western Atlantic and any site in the Indo-Pacific, with Φ_ST_ values ranging from 0.859 to 0.976 (Table 3). The high genetic structure and reciprocal monophyly together indicate a prolonged period with no gene flow between these ocean basins, and this inference is further supported by our TMRCA dates (Fig. 2). Below we discuss evidence for the distinction of the Atlantic and Pacific lineages, and potential historical scenarios that could have led to their divergence.

### Phylogenetic differentiation and taxonomic status

Various sequence divergence levels have been proposed as thresholds to delineate species of marine invertebrates, but a consensus value has not been reached (Hickerson, Meyer & Moritz, 2006; Matzen da Silva et al., 2011; Meier, Zhang & Ali, 2008; Quan et al., 2004). For decapods, within-species COI sequence divergences range from 0.24 to 1.8% (Iacchei et al., 2016; Ketmaier, Argano & Caccone, 2003; Knowlton & Weigt, 1998; Matzen da Silva et al., 2011; Morrison, Ríos & Duffy, 2004; Quan et al., 2004), while divergence among species within a genus are typically higher (2.5%–32.7%; Matzen da Silva et al., 2011). For *S. hispidus*, we observed a sequence divergence of 2.1% between the Western Atlantic and Indo-Pacific lineages, which falls in the grey zone of divergences-within versus divergences-between species. We observed a broad range of pairwise differences between congeners within our *Stenopus* phylogeny from 4 to 14%, based on four of the 11 recognized species. Specifically, we calculated a divergence of 4% between *S. hispidus* and *S. pyrsonotus*, 10.6% between *S. hispidus* and *S. zanzibaricus*, and 14% between *S. hispidus* and *S. scutellatus*. Without data from the remaining seven species within the genus, it is unclear how the 2.1% divergence compares to other divergences in the genus*.* However, high pairwise Φ_ST_ values and no shared haplotypes between the Western Atlantic and Indo-Pacific clades invokes the question of whether these lineages are evolutionary distinct units below the species level (Moritz, 1994), or whether the distinction is sufficient for formal taxonomic recognition. Chen et al. (2016) found that *Stenopus* is a polyphyletic genus and at least two species, *S. earlei* and *S. goyi*, should be reclassified into a separate genus *Juxtastenopus*. Combined with our findings here, a taxonomic re-evaluation of the genus is clearly justified, along with additional taxon sampling to determine species boundaries within the genus *Stenopus*.

Unexpectedly, we observed much higher genetic diversity in the Western Atlantic *S. hispidus* population (*h* = 0.929) than in the Indo-Pacific populations (*h* = 0.165). This result is striking, because the expectation is that the habitats in the Indo-Pacific (>18,000 km) should support a larger population size, and in turn, much greater genetic diversity than that of the more homogeneous and smaller (∼2,000 km) Western Atlantic. Lower diversity in the Indo-Pacific could result from such factors as abundance, population structuring, sex ratios, or mating architecture, but none of these would result in a single common haplotype shared across the Red Sea, Indian and Pacific Oceans. There are two classical explanations for the lack of genetic diversity in one population relative to another. First, the Indo-Pacific population may have experienced a recent bottleneck or selective sweep, reducing the genetic variation in that population (Nei, Maruyama & Chakraborty, 1975). In this scenario, either a single mtDNA haplotype was selectively favored or only a single haplotype survived a bottleneck. To account for the observed data, a bottleneck would need to be so severe or so long that across >18,000 km only a single haplotype remains (which simulations suggest would require 30 or fewer individuals for 20 generations under random mating—Hoelzel et al., 1993). This hypothetical bottleneck did not similarly impact any other species studied to date, and did not affect the much smaller and more homogeneous Atlantic Ocean. Subsequently, that tiny bottlenecked population would have to rebound and spread across more than half of the planet so recently that this single haplotype has not diversified throughout the sampling range. Both of these components for the first mechanism seem highly unlikely, and Bayesian skyline plots suggest a stable Atlantic population and a recent population expansion in the Indo-Pacific rather than a bottleneck (Fig. S2). Second, the low genetic diversity throughout the Red Sea and Indo-Pacific may be due to a relatively recent colonization of the Indian and Pacific Oceans, that would either have to come from some refugium (which again invokes a population crash), or from the Atlantic, the only known source population. This colonization and expansion explanation is equally unsatisfying because if this had occurred, we would expect to see shared haplotypes between the Western Atlantic and Indo-Pacific, which is not the case (Fig. 3). Instead, the two populations are reciprocally monophyletic, meaning either the haplotype that colonized the Indo-Pacific is sufficiently rare in the Western Atlantic that we did not detect it, or the time since colonization is long enough that lineage sorting has occurred, but not long enough for more diverse haplotypes to have become established in the Pacific. Coalescent analysis reveals the TMRCA in *S. hispidus* is roughly 1.2 Mya, with the extant Western Atlantic population estimated to be ∼500,000 years old (95% HPD = 300,000–760,000 yr), and the extant Indo-Pacific population ∼200,000 years old (95% HPD = 60,000–400,000 yr). All else being equal, given a split between Western Atlantic and Indo-Pacific lineages, we would expect lower rates of haplotype loss, and greater haplotype diversification, within the vast Indo-Pacific and Red Sea than the much smaller Atlantic over the same timescale. Additional studies with nuclear markers and expanded sampling throughout the Indo-Pacific may shed light on this unexpected finding.

### Phylogeography

To understand the patterns in regional diversification of *S. hispidus*, we examined a few historical biogeographic scenarios that may explain the phylogeographic relationships observed in *S. hispidus*. Given our TMRCA dates of the lineage split (Fig. 2), there are two plausible phylogeographic hypotheses to explain the divergence of the Western Atlantic and Indo-Pacific lineages: (1) the closure of the Isthmus of Panama, and (2) dispersal between the Western Atlantic, Red Sea, Indian and Pacific Oceans, each discussed below.

### Closure of the Isthmus of Panama

The closure of the Isthmus of Panama (∼3 Mya; O’Dea et al., 2016) formed a virtually impenetrable land barrier between the Eastern Pacific and the topical Western Atlantic, significantly altering the physical and oceanographic environments of both oceans (D’Croz & Robertson, 1997). Tropical marine fauna became isolated on either side of Panama as the Isthmus shoaled prior to the final closure (Lessios, 2008), leading to present day divergences in a variety of marine taxa (Knowlton & Weigt, 1998; Lessios, 2008; Marko & Moran, 2009; Marko, Eytan & Knowlton, 2015).

*Stenopus hispidus* is not well established in the Eastern Tropical Pacific (ETP) (H Lessios, pers. comm., 2015) but has been regularly documented as far east in the Pacific Ocean as Easter Island, Chile (De los Ríos Escalante & Arancibia, 2016; Poupin, 2003; Retamal & Moyano, 2010). To date, however, the only known record of *S. hispidus* in the ETP is a single individual collected from Taboga Island, Panama during a 1916 expedition (Goy, 1987). Rare individuals possibly occur as strays from the Central Pacific, but the absence of any documentation of this colorful shrimp valued by the aquarium trade indicates a sustainable ETP population likely does not exist, possibly due to inadequate habitat. The closure of the Isthmus had dramatic effects on the marine fauna in the Pacific, such that the ETP experienced a decline among planktonic communities, large shifts in oceanographic conditions, and the loss of ETP coral reefs, resulting in greatly reduced habitat for many species (reviewed by Leigh, O’Dea & Vermeij, 2014; O’Dea et al., 2016). A more recent collapse of reef ecosystems in the ETP occurred ∼4,000 years ago due to increasing El Niño–Southern Oscillation (ENSO) variability, with reef decline continuing for ∼2,500 years (Toth et al., 2012). Eastern Tropical Pacific reef assemblages are also influenced by strong wind jets that cross the Isthmus of Panama into the Pacific region and cause seasonal upwelling of cold, nutrient-rich waters (Xie et al., 2005). Similar unsuitable conditions may also explain the absence of *S. hispidus* where upwelling predominates along the western coast of Africa (Limbaugh, Pederson & Chace, 1961), despite the presence of *S. hispidus* populations in adjacent areas on either side of the upwelling zone. Although *S. hispidus* is a broadly distributed (both geographically and among depth zones), it is possible that initial colonists of the ETP are wiped out due to a loss of suitable habitat in that region.

Regardless, our estimates of divergence between the Western Atlantic and Pacific are inconsistent with the Isthmus of Panama closure being the ultimate mechanism responsible for the observed divergence. The TMRCA between the Western Atlantic and the Indo-Pacific lineages in *S. hispidus* was estimated at 1.2 Mya (95% HPD = 710,000–1.8 Mya) (Fig. 2). Even the oldest 95% HPD estimated date for the lineage split (∼1.8 Mya) occurred at least a million years after the final closure of the Isthmus (O’Dea et al., 2016), eliminating the closure of the Isthmus as a likely source of the separation of the Western Atlantic and Pacific lineages.

### Benguela and Agulhas Currents

An alternative hypothesis for the divergence in *S. hispidus* lineages is a rare dispersal event via the currents around South Africa. Currents at the southern tip of Africa have facilitated species dispersal between the Atlantic and Indian Oceans (Gaither et al., 2015b; Garber, Tringali & Franks, 2005; Portnoy et al., 2015), maintaining connections of tropical marine organisms around the globe despite the closure of both the Tethys seaway and the Central American seaway. The two predominant currents in this region are the cold Benguela Current moving northward along the west coast of Africa, and the warm Agulhas Current moving southward along the east coast.

The cool, northward flowing Benguela Current results in cold water upwelling near the coast and creates a dispersal barrier for most tropical organisms. Even marine species with large pelagic adults, such as the whale shark, *Rhincodon typus* (Castro et al., 2007), blue marlin, *Makaira nigricans* (Buonaccorsi, McDowell & Graves, 2001) and the deep-dwelling Escolar, *Lepidocybium flavobrunneum* (Brendtro, McDowell & Graves, 2008) have genetically distinct populations on either side of the Benguela Barrier, although some other oceanic migrants, including wahoo, *Acanthocybium solandri* (Garber, Tringali & Franks, 2005; Theisen et al., 2008), Mahi-mahi, *Coryphaena hippurus* (Diaz-Jaimes et al., 2010), and skipjack tuna, *Katsuwonus pelamis* (Graves, Ferris & Dizon, 1984; Ely et al., 2005) reveal no significant population structure across this barrier.

Among the non-pelagic species that have successfully crossed the Benguela Current, most have been aided by the warm Agulhas Current moving southward along the east coast of Africa. The Agulhas is a fast-flowing, warm-water current supplying Indian Ocean water into the Atlantic Ocean (Shannon, 1970), and concurrently aiding in the dispersal of tropical organisms from the Western Indian Ocean province into the Atlantic. Agulhas rings are formed, providing a pulse of warm and salty water advected through the Benguela Current from the Indian Ocean into the Atlantic (Hutchings et al., 2009). Genetic studies have shown colonization from the Indian into the Atlantic Ocean for the marlin sucker, *Remora osteochir* (Gray et al., 2009), sandbar shark, *Carcharhinus plumbeus* (Portnoy et al., 2010), Atlantic goldspot goby, *Gnatholepis thompsoni* (Rocha et al., 2005), and the Atlantic pygmy angelfishes (genus *Centropyge*) (Bowen et al., 2006). If *S. hispidus* had followed this path, we would have to explain how the Atlantic has become more diverse and with an older, more stable population than the entire Red Sea, Indian and Pacific Oceans—a pattern not seen in any of the other species reviewed here.

Colonizing in the opposite direction, from the Atlantic Ocean to the Indian Ocean is rare, particularly for tropical marine species without large pelagic adults. The glasseye fish, *Heteropriacanthus cruentatus* is one of few examples of a non-pelagic tropical species that has dispersed through the Benguela Current from the Atlantic into the Indian Ocean, with two divergent lineages (a Caribbean lineage and a widely distributed Indo-Pacific lineage) separated by 10% at COI (Gaither et al., 2015b). However, this event is inferred to have happened much earlier (∼15 Mya). Although clearly not concurrent events, because our coalescent analyses indicate that Western Atlantic and Indo-Pacific *S. hispidus* shared a common ancestor after the closure of the Isthmus of Panama, it is possible that a similar rare dispersal event through the Benguela Current is the origin of our observed divergence. The coalescent estimates of TMRCA indicate that the Indo-Pacific population has more recently expanded than the Atlantic, and thus, it is possible that a one-time dispersal event occurred from the more diverse Western Atlantic around South Africa to colonize the Indian and Pacific Oceans. However, there are no shared haplotypes, and this line of evidence would be either discounted or greatly strengthened with the addition of specimens from the Eastern Atlantic (West Coast of Africa), where reports of *S. hispidus* are rare, perhaps for the same reasons as in the ETP. If this most common Indo-Pacific haplotype also occurs in the Eastern Atlantic, *S. hispidus* would be the only tropical invertebrate species reported to date to follow this surprising colonization route and disperse eastward through the cold Benguela Current.

### Genetic structure within the Indo-Pacific

In addition to differences in the rate of haplotype loss or diversification and lack of shared haplotypes between the Western Atlantic and Indo-Pacific lineages of *S. hispidus*, there is also significant population structure across our Indo-Pacific sampling locations, spanning from the Red Sea to French Polynesia and Hawai‘i. This broad region extends over approximately half the circumference of the globe, and contains six tropical biogeographic provinces (Briggs & Bowen, 2012; Toonen et al., 2016). *Stenopus hispidus* occurs in five of these six provinces, and we obtained samples from all five: the Red Sea Province, Western Indian Ocean, Indo-Polynesian Province, the Hawaiian Islands, and Marquesas, although in some locations the species is rare, limiting our collections to low sample sizes. Across this Indo-Pacific range, *S. hispidus* shows significant population structure among some of the peripherally sampled locations: the Red Sea and Hawai‘i (Φ_ST_ = 0.189, *P* < 0.001), and the Red Sea and Palmyra Atoll (Φ_ST_ = 0.135, *P* < 0.009). However, *S. hispidus* does not show significant structure between the Red Sea and French Polynesia, which included individuals from Marquesas (Φ_ST_ = 0.070, *P* < 0.272), nor among any of the non-Red Sea comparisons throughout this broad geographic area (Table 3). The small sample size and limited sampling across many intermediate locations precludes strong conclusions about the scale of dispersal and the population structure within ocean basins. Nevertheless, given the significant population structure among the well-sampled extremes across the Indo-Pacific, *S. hispidus* clearly does not maintain a single population that spans this region.

Our data is consistent with the growing database that supports the pattern of peripheral isolation among reef fishes and invertebrates distributed across the Indo-Pacific region (Bernardi et al., 2004; Budd & Pandolfi, 2010; Fernandez-Silva et al., 2015; Hodge et al., 2012; Iacchei et al., 2016). Provinces on the periphery of the Indo-Pacific host both distinct populations and distinct species of more broadly distributed taxa (reviewed by Bowen et al., 2016; DiBattista et al., 2015; Gaither et al., 2011), and are known to be hotspots of endemism for a variety of taxa (Kay, 1980; Abbott, 1999; Randall, 2007; DiBattista et al., 2016). Our data most strongly support the isolation of the Red Sea population of *S. hispidus* from the rest of the Indo-Pacific region. The Red Sea shows high levels of endemism in a variety of marine phyla (DiBattista et al., 2016), and many of the endemic fish species have sister taxa in the Western Indian Ocean whose geographic ranges span the Indo-Pacific (DiBattista et al., 2015). The evolutionary history of the Red Sea is much more complex than known previously, and in addition to simply isolating individuals of widespread taxa that eventually evolve into Red Sea endemics, recent evidence indicates the Red Sea has the potential to export biodiversity as well (Coleman et al., 2016; DiBattista et al., 2013; DiBattista et al., 2016). In the case of *S. hispidus,* although the genetic differentiation between the Red Sea and the Indo-Pacific region is relatively high (Φ_CT_ = 0.235, *P* = 0.029), we observed both haplotypes that were private to the Red Sea, as well as a common haplotype that was shared across the breadth of the Indo-Pacific. This pattern may indicate incomplete lineage sorting in the Red Sea population (which would implicate the Red Sea as the refuge from which the Indo-Pacific was recolonized), or a more recent colonization by the Indo-Pacific lineage into a Red Sea population formerly isolated by Pleistocene sea level fluctuations. In either case, the significant population structure suggests there is likely limited gene flow occurring between these provinces, and additional study in this portion of the range is likely to offer fruitful insights.

## Conclusions

Here, we provide the first range-wide phylogeographic study of the banded coral shrimp *S. hispidus*, extending across more than 27,000 km of the globe. Despite having a circumtropical distribution and a ∼253 day PLD, one of the longest mean larval durations in the marine realm, our data shows that gene flow is not sufficient to homogenize populations across the known species range. The recovery of two distinct lineages in the Western Atlantic and Indo-Pacific calls into question whether populations in these ocean basins are in fact a single taxonomic unit. Coalescence estimates indicate the closure of the Isthmus of Panama predated the divergence between extant lineages in the Western Atlantic and Indo-Pacific by more than a million years, eliminating closure of the Isthmus as a likely isolating mechanism. Although no explanation for the observed pattern is entirely satisfactory, the weight of available evidence points to the Indo-Pacific lineage arising by a rare dispersal event from the Atlantic around South Africa via the Benguela Current, thereby colonizing the Indian and Pacific Oceans, followed by dispersal across the Indo-Pacific in the last 200,000 years. Surprisingly, this benthic coral reef associated shrimp shows a single haplotype dominating the largest continuous tropical oceanic expanse on the planet—a result not seen in any other globally distributed marine species, including such highly mobile species as whale sharks and tunas (Theisen et al., 2008). This surprising finding challenges our understanding of the genetic architecture for species with high pelagic dispersal potential and circumtropical distributions. Further taxonomic and geographic sampling, particularly across the Red Sea, Indian and Eastern Atlantic Oceans, and inclusion of many additional loci to these comparisons may shed new light on this unexpected finding. From the present study, we know this is the little shrimp that could defy expectations, by apparently colonizing against the Benguela Current, and dispersing across an Indo-Pacific range that spans more than half the planet.

##  Supplemental Information

10.7717/peerj.4409/supp-1 Figure S1 A Bayesian time-calibrated phylogeny of *Stenopus hispidus* A Bayesian time-calibrated phylogeny based on the cytochrome *c* oxidase subunit I (COI) of *Stenopus hispidus* with sister species (*S. pyrsonotus*, *S. zanzibaricus, S. scutellatus*) and sister genera (*Synalpheus* sp. and *Alpheus bellulus*)** as outgroups (Shi et al., 2012) (525 bp) was generated using BEAST. The Western Atlantic lineage of *S. hispidus* is shown in green (*N* = 50; Curaçao, Belize, and Panama). The Indo-Pacific lineage of *S. hispidus* is shown in blue (*N* = 148; Hawai‘i, Palmyra Atoll, French Polynesia, Philippines, Maldives, Tanzania, and the Red Sea). Values above nodes are median node ages with 95% HPD intervals represented by blue node bars.

10.7717/peerj.4409/supp-2 Figure S2 A Bayesian Skyline Plot (A) The Western Atlantic shows a relatively stable population whereas the (B) Overall Dataset with the Pacific population included shows evidence of a recent population expansion.

10.7717/peerj.4409/supp-3 Supplemental Information 1 Unique sequences of *Stenopus hispidus* FASTA file of unique sequences for *Stenopus hispidus.*

## Additional Information and Declarations

### Field Study Permissions

The following information was supplied relating to field study approvals (i.e., approving body and any reference numbers): Field experiments were approved by Papahānaumokuākea Marine National Monument (PMNM) with collection permit no. PMNM-2008-047, PMNM-2009-032, PMNM-2010-037, PMNM-2011-041, PMNM-2012-049).

### Data Availability

The following information was supplied regarding data availability: GeOMe: http://www.geome-db.org/query. Search: *Stenopus hispidus*. Genbank MG819599–MG819631.
