# Supplementary figures and images for: The little shrimp that could: phylogeography of the circumtropical Stenopus hispidus (Crustacea: Decapoda), reveals divergent Atlantic and Pacific lineages
Source: PeerJ. 2018 Mar 6;6:e4409. doi: 10.7717/peerj.4409 (PMC5844259; doi:10.7717/peerj.4409)

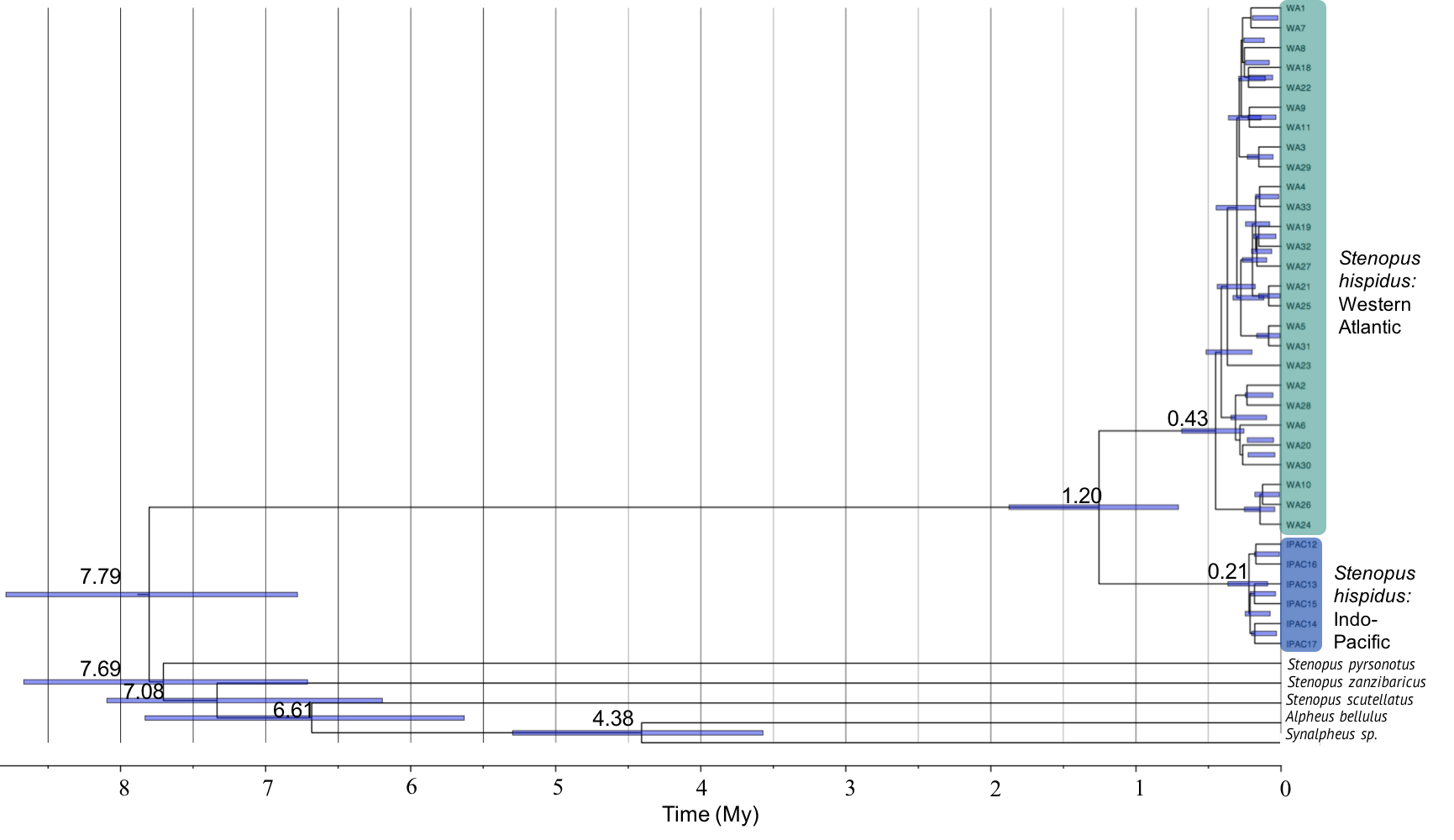

Supplement: Figure S1 — A Bayesian time-calibrated phylogeny based on the cytochrome c oxidase subunit I (COI) of Stenopus hispidus with sister species (S. pyrsonotus, S. zanzibaricus, S. scutellatus) and sister genera (Synalpheus sp. and Alpheus bellulus) as outgroups (Shi et al., 2012) (525 bp) was generated using BEAST. The Western Atlantic lineage of S. hispidus is shown in green (N = 50; Curaçao, Belize, and Panama). The Indo-Pacific lineage of S. hispidus is shown in blue (N = 148; Hawai‘i, Palmyra Atoll, French Polynesia, Philippines, Maldives, Tanzania, and the Red Sea). Values above nodes are median node ages with 95% HPD intervals represented by blue node bars. [file peerj-06-4409-s001.png]

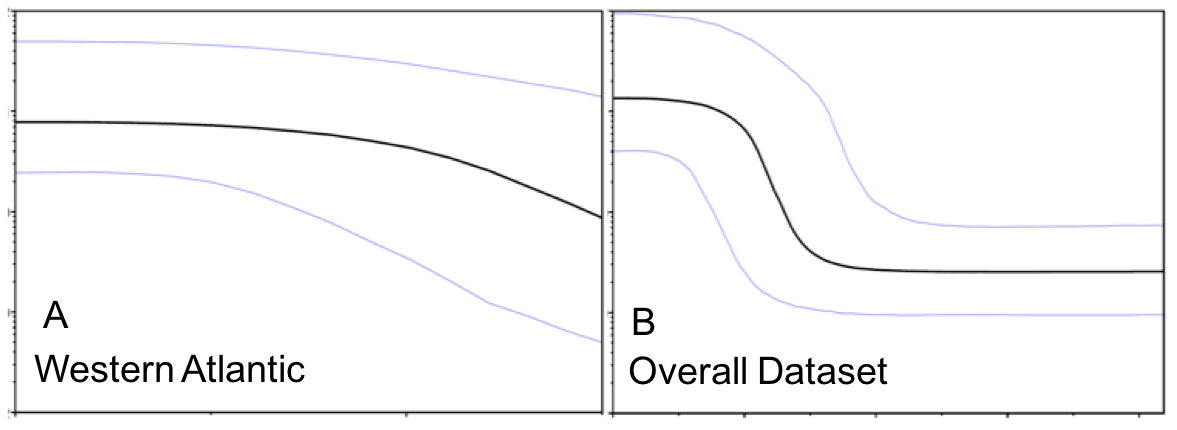

Supplement: Figure S2 — (A) The Western Atlantic shows a relatively stable population whereas the (B) Overall Dataset with the Pacific population included shows evidence of a recent population expansion. [file peerj-06-4409-s002.png]
